# Timing of eye removal influences low-vision quality of life and self-perception of facial appearance in people with one eye

**DOI:** 10.1371/journal.pone.0323603

**Published:** 2025-05-15

**Authors:** Aysha N. Kinakool, Stefania S. Moro, Jennifer K. E. Steeves

**Affiliations:** Department of Psychology, Centre for Vision Research, and Centre for Integrative and Applied Neuroscience, York University, Toronto, Canada; Mansoura University Faculty of Veterinary Medicine, EGYPT

## Abstract

**Purpose:**

The loss of one eye early compared to late in life coincides with critical periods of postnatal visual development and is associated with sensory adaptations which may lead to different psychosocial experiences throughout life. This study investigates the impact of age at enucleation on psychosocial health factors, namely low-vision quality of life, self-perception of facial appearance, anxiety, and depression.

**Methods:**

Twenty-two participants with early or late eye removal and twenty-seven binocular viewing controls completed the Low-Vision Quality of Life questionnaire, Facial Appearance sub-scale of the Negative Physical Self-Scale and the Hospital Anxiety and Depression scale.

**Results:**

Low-vision quality of life was lower for people who had their eye removed late in life compared to binocular viewing controls. Further, within this group, lower self-perception of facial appearance was associated with lower low-vision quality of life. Moreover, experience with binocularity and time since enucleation were not associated with psychosocial health factors.

**Conclusion:**

Developmental factors rather than experiential factors appear to influence psychosocial health since binocular experience and time since enucleation were not related to any psychosocial factor. These findings suggest that people who have had their eye removed early in life, during the critical periods of visual development may benefit from better psychosocial outcomes.

## Introduction

At birth, the visual system is physiologically and functionally not fully developed and balanced binocular input allows for typical postnatal maturation [1]. Multiple overlapping critical periods exist that determine the development of typical visual function and visual maturation can extend into our third decade of life for some functions (e.g., face recognition) [2]. Disruption of visual input during this maturation period can lead to anatomical changes along different structures in the visual pathway, changes in cortical function, as well as changes in visual and audiovisual behaviour [1]. The timing of visual disruption can have differential effects on visual maturation.

A unique model for examining the consequences of the loss of binocularity during the critical periods of visual development is unilateral eye enucleation [3]. Unilateral eye enucleation is the surgical removal of one eye due to, for example ocular malignancy, traumatic injury, or eye disease [4]. This yields complete deafferentation of one channel of visual input to the brain unlike other forms of partial visual deprivation such amblyopia which frequently has remaining anomalous visual signals from the affected eye [3,5]. There is a growing body of behavioural and neuroimaging research showing physiological and functional adaptations that accommodate when one eye is lost early in life (e.g., reviewed in [3,5]). Early eye enucleation has been shown to be associated with changes in subcortical and cortical structures [6], visual, auditory, and audiovisual brain function and behaviour [6–12].

These adaptations do not appear when one eye is removed late in life. For example, contrast sensitivity shows enhancements for early but not late eye enucleation [13], indicating a developmental relationship between visual ability and the timing of the loss of one eye. Removing one eye late in life is associated with more reduction in volume compared to losing one eye early in life in the lateral geniculate nucleus (the visual relay centre of the thalamus) [7]. Adaptations in other sensory systems have also been observed across sensory modalities. People who have had one eye removed early in life have reduced susceptibility to audiovisual illusions such as the McGurk effect [8–11] unlike those who had one eye removed late in life and binocular viewing control participants [14]. This indicates that binocular experience and its relation to critical periods of visual development influences cortical reorganization and behaviour differently in people who have had one eye removed early compared to late in life. Considering the differential effects of early compared to late eye enucleation, it is plausible that timing of eye enucleation not only impacts the brain and its sensory abilities but could also influence psychosocial visual experience with the world and ultimately quality of life.

Previous research has shown that removing an eye is related to changes in psychosocial experience and quality of life. While some studies have shown people who have had an eye removed have higher or a similar quality of life to healthy controls [15–18], there are also reports of lower quality of life [19–22]. Moreover, people with one eye have reported feelings of social anxiety and avoid social situations associated with their altered appearance and negative feelings about how they are viewed [23]. This avoidance and social anxiety can lead to feelings of anger, depression, and isolation [24]. Eye enucleation in general is associated with increased anxiety and depression and decreased positive self-perception of facial appearance [25–27]. For instance, higher levels of anxiety and depression are associated with lower vision-related quality of life and with greater concerns about facial appearance [27,28]. Higher anxiety is associated with decreased appearance related social function but is not associated with vision-related quality of life [25]. Some have found anxiety and depression levels are elevated but nonetheless within the typical range for the general population [23] while others have found increased prevalence of anxiety and depression compared to the general population [29]. The variability across studies may be a reflection of differences between having one’s eye removed early compared to late in life.

Since there is some evidence that removing one eye is related to changes in psychosocial factors and that the timing of visual disruption can have differential effects on visual system maturation, it is plausible that timing of enucleation may be an important factor in determining psychosocial outcomes. The aim of this study was to investigate the impact of age at enucleation and psychosocial health, namely low-vision quality of life (LVQoL), self-perception of facial appearance, anxiety, and depression. We hypothesized that because adults who have had their eye removed early in life have experienced different physiological and behavioural adaptions compared to adults who have had their eye removed late in life, these adaptations may be related to a different psychosocial trajectory.

## Methods

### Participants

#### Participants with early monocular enucleation (E-ME).

Thirteen adult participants who had undergone early monocular enucleation participated, mean (SD) = 45 (10) years. All E-ME participants had undergone unilateral eye enucleation (8 right and 5 left eye) due to retinoblastoma (a childhood cancer of the retina, n = 11), traumatic injury (n = 1), or eye disease (n = 1). Age at enucleation ranged from 12–60 months, mean (SD) = 20 (13) months.

#### Participants with late monocular enucleation (L-ME).

Nine adult participants who had undergone late monocular eye enucleation participated, mean (SD) = 51 (10) years. All L-ME participants had been unilaterally enucleated (2 right eye and 7 left eye) due to ocular malignancy (n = 1), traumatic injury (n = 7), or other (n = 1). Age at enucleation ranged from 84–744 months, mean (SD) = 335 (223) months.

#### Binocular viewing participants (BV).

The control group consisted of twenty-seven binocularly intact participants, mean (SD) = 47 (11) years.

Participant recruitment and data collection were conducted in three testing intervals – 1^st^ November 2021–13^th^ February 2022; 14^th^ April 2022–16^th^ January 2023; and 7^th^ November 2023–1^st^ January 2024. Written consent was obtained from all participants. All participants (E-ME, L-ME, and BV) reported normal or corrected-to-normal vision (in the remaining eye for participants with one eye). Participants were excluded if they had any other eye diseases, psychiatric, or neurological conditions. The study was approved by the York University Office of Research Ethics (Ethics Certificate: e2021-085) and adhered to the tenets of the Declaration of Helsinki. Participant demographics are presented in Table 1.

**Table 1:** Participant Demographics.

Participant ID	Group	Age (years)	Sex	Ethnicity	Eye Removed	Age at Enucleation (months)
E001	E-ME	37	F	White	Left	12
E002	E-ME	48	F	Asian	Left	12
E003	E-ME	34	F	White	Right	12
E004	E-ME	51	M	White/ South Asian	Left	24
E005	E-ME	41	M	White	Left	12
E006	E-ME	40	F	White	Right	12
E007	E-ME	42	M	White	Left	12
E008	E-ME	56	F	White	Right	24
E009	E-ME	56	F	White	Right	24
E010	E-ME	37	F	White	Right	24
E011	E-ME	41	M	White	Right	12
E012	E-ME	68	M	White	Right	60
E013	E-ME	34	M	White	Right	24
L001	L-ME	53	F	White/ Asian	Right	624
L002	L-ME	62	F	White	Left	744
L003	L-ME	59	F	White	Left	312
L004	L-ME	55	F	White	Left	84
L005	L-ME	49	M	West Asian	Left	360
L006	L-ME	56	M	White	Left	144
L007	L-ME	58	M	White	Left	216
L008	L-ME	31	F	White	Right	372
L009	L-ME	39	F	White	Left	156
C001	BV	34	M	South Asian	NA	NA
C002	BV	30	F	South Asian	NA	NA
C003	BV	61	M	South Asian	NA	NA
C004	BV	38	F	South Asian	NA	NA
C005	BV	59	F	White	NA	NA
C006	BV	58	M	White	NA	NA
C007	BV	50	F	White	NA	NA
C008	BV	61	F	White	NA	NA
C009	BV	66	F	White	NA	NA
C010	BV	57	F	White	NA	NA
C011	BV	59	F	White	NA	NA
C012	BV	56	F	White	NA	NA
C013	BV	32	M	White	NA	NA
C014	BV	55	F	White	NA	NA
C015	BV	42	M	White	NA	NA
C016	BV	42	F	White	NA	NA
C017	BV	57	F	White	NA	NA
C018	BV	43	M	White	NA	NA
C019	BV	32	M	South Asian	NA	NA
C020	BV	37	M	South Asian	NA	NA
C021	BV	48	F	Mixed	NA	NA
C022	BV	41	M	White	NA	NA
C023	BV	36	F	South Asian	NA	NA
C024	BV	54	M	White	NA	NA
C025	BV	37	M	Mixed	NA	NA
C026	BV	32	M	White	NA	NA
C027	BV	52	M	White	NA	NA

E-ME: Early Monocular Enucleation; L-ME: Late Monocular Enucleation; BV: Binocular Viewing.

### Procedure

A self-administered cross-sectional online survey was presented using Qualtrics (Qualtrics XM, Provo, Utah, USA). Four indicators related to psychosocial health were measured: low-vision quality of life (LVQoL), self-perception of facial appearance, anxiety, and depression. LVQoL was assessed using the Low-Vision Quality of Life questionnaire which examines four areas: 1. Distance vision, mobility, and lighting; 2. Adjustments; 3. Reading and fine work; and 4. Activities of daily living [30]. A higher LVQoL score indicates that an individual has a higher quality of life in relation to their vision. Self-perception of facial appearance was assessed using the Facial Appearance sub-scale of the Negative Physical Self-Scale where higher scores indicate a higher self-perception of one’s facial appearance [31]. Finally, anxiety and depression were assessed using the Hospital Anxiety and Depression scale where higher scores indicate higher levels of anxiety and depression [32].

## Results

### Psychosocial factors across groups

**Fig 1:**
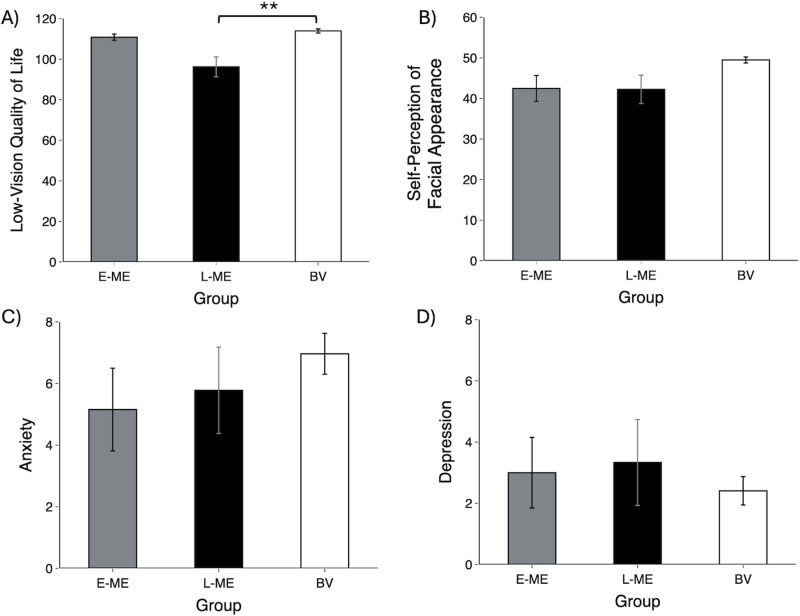
Mean scores of psychosocial factors across groups; A) Low-vision quality of life (LVQoL), B) Self-perception of facial appearance, C) Anxiety, and D) Depression. LVQoL of L-ME is lower than BV group. Error bars represent standard error of the mean (SEM). E-ME = Participants with early enucleation (grey), L-ME = Participants with late enucleation (black), BV = Binocular viewing (white). ** p < 0.01.

Non-parametric Kruskal Wallis tests were conducted for non-normal data between groups (E-ME, L-ME, BV) for each psychosocial factor (LVQoL, self-perception of facial appearance, anxiety, and depression). Group differences were found for LVQoL, χ^2^ (2, 49) = 14.38, p = 0.001, η_p_^2^ = 0.230. Post-hoc pairwise comparisons using Dunn-Bonferroni correction indicated LVQoL for L-ME was lower compared to BV controls (p = 0.001) (Fig 1A). There was no difference in self-perception of facial appearance, χ^2^ (2, 49) = 3.48, p = 0.176, η_p_^2^ = 0.068 (Fig 1B). There was no difference in anxiety scores, χ^2^ (2, 49) = 3.26, p = 0.196, η_p_^2^ = 0.064 (Fig 1C). Finally, there was no difference in depression scores,χ^2^ (2, 49) = 0.13, p = 0.936, η_p_^2^ = 0.003 (Fig 1D). Fig 1 illustrates the group differences for each psychosocial factor.

### Self-perception of facial appearance and psychosocial factors

**Fig 2:**
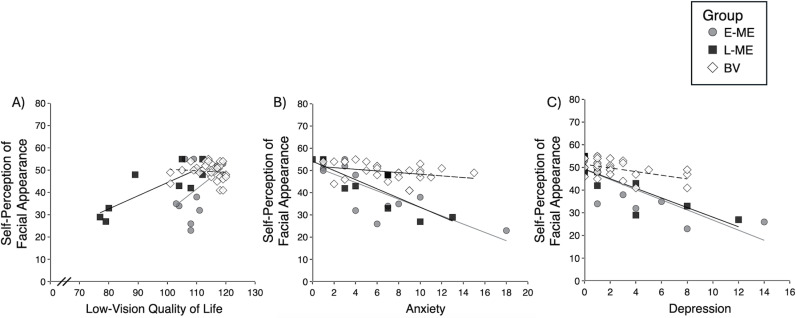
Scatterplots depicting the relation between self-perception of facial appearance and A) Low-vision quality of life (measured using LVQoL), B) Anxiety (measured using Hospital Anxiety and Depression Questionnaire), and C) Depression (measured using Hospital Anxiety and Depression Questionnaire). E-ME = Participants with early enucleation (grey circles), L-ME = Participants with late enucleation (black squares), BV = Binocular viewing (white diamonds).

Spearman correlations were conducted comparing psychosocial factors (LVQoL, anxiety, and depression) and self-perception of facial appearance for each group (E-ME, L-ME, BV). There was a strong positive correlation where lower self-perception of facial appearance was associated with lower LVQoL in the L-ME group, r_s_(7) = 0.77, p = 0.016 (Fig 2A). There was no relation between self-perception of facial appearance and LVQoL for E-ME, r_s_(11) = 0.32, p = 0.281 or BV controls, r_s_(25) = -0.26, p = 0.188.

Spearman correlations were conducted comparing self-perception of facial appearance and anxiety and indicated an inverse relation where low anxiety was associated with high self-perception of facial appearance in the L-ME (r_s_(7) = -0.78, p = 0.014), E-ME (r_s_(11) = -0.78, p = 0.002), and BV groups (r_s_(25) = -0.38, p = 0.050) (Fig 2B).

Finally, Spearman correlations were conducted comparing self-perception of facial appearance and depression and indicated a strong inverse relation where low depression was associated with high self-perception of facial appearance in the L-ME (r_s_(7) = -0.90, p < 0.001), E-ME (r_s_(11) = -0.75, p = 0.003), and BV groups (r_s_(25) = -0.48, p = 0.010) (Fig 2C).

### Binocular experience and psychosocial factors

In order to explore whether binocular experience, independent of age, impacts psychosocial factors we examined the relation between the number of months a person lived with binocular vision (before monocular enucleation) and psychosocial factors (LVQoL, self-perception of facial appearance, anxiety, and depression). A Mann Whitney U test comparing binocular experience between monocular enucleation groups (E-ME and L-ME) indicated that the E-ME group had less binocular experience compared to the L-ME group, *U* = -91, p < 0.001, r = -2.13. Binocular viewing control participants were not included in these analyses as binocular experience is simply a reflection of their age.

**Fig 3:**
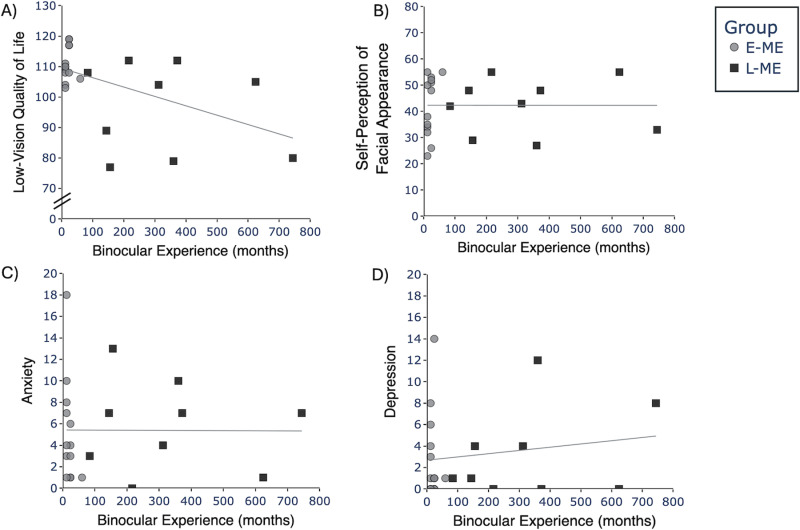
Scatterplots depicting the relation between binocular experience and **A)** Low-vision quality of life (LVQoL), **B)** Self-perception of facial appearance, **C)** Anxiety, and **D)** Depression across groups. E-ME = Participants with early enucleation (grey circles), L-ME = Participants with late enucleation (black squares).

Spearman correlation analyses were conducted to investigate the relation between binocular experience and psychosocial factors (LVQoL, self-perception of facial appearance, anxiety, and depression). There was no relation between binocular experience and LVQoL, r_s_(20) = -0.32, p = 0.150 (Fig 3A); self-perception of facial appearance, r_s_(20) = 0.12, p = 0.593 (Fig 3B); anxiety, r_s_(20) = -0.08, p = 0.739 (Fig 3C); or depression, r_s_(20) = -0.01, p = 0.956 (Fig 3D).

### Time since enucleation and psychosocial factors

In order to explore whether time since enucleation impacts psychosocial factors, we examined the relation between the number of months a person has lived since enucleation and psychosocial factors (LVQoL, self-perception of facial appearance, anxiety, and depression). A Mann Whitney U test indicated that the E-ME group lived a longer time since enucleation compared to the L-ME group, *U* = 2, p = 0.023, r = -0.80.

**Fig 4:**
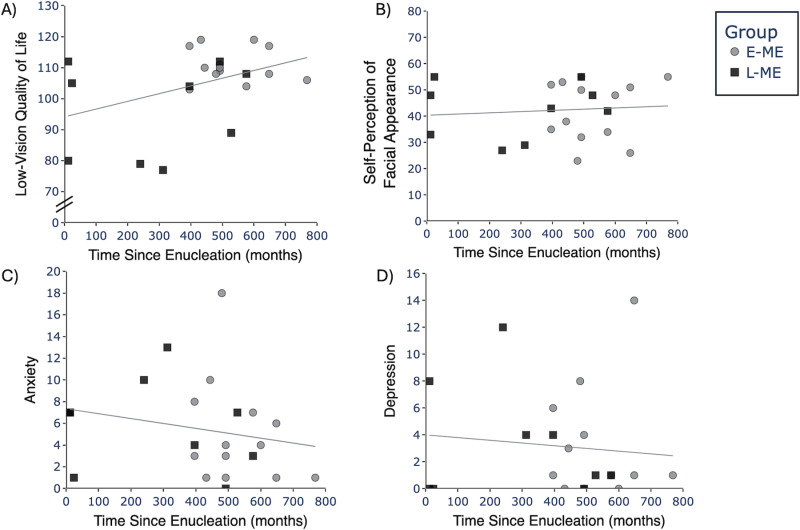
Scatterplots depicting the relation between time since enucleation and **A)** Low-vision quality of life (LVQoL), **B)** Self-perception of facial appearance, **C)** Anxiety, and **D)** Depression. E-ME = Participants with early enucleation (grey circles), L-ME = Participants with late enucleation (black squares).

Spearman correlation analyses showed no relation between time since enucleation and LVQoL, r_s_(20) = 0.30, p = 0.181 (Fig 4A); self-perception of facial appearance, r_s_(20) = 0.14, p = 0.532 (Fig 4B); anxiety, r_s_(20) = -0.35, p = 0.108 (Fig 4C); or depression, r_s_(20) = -0.13, p = 0.571 (Fig 4D).

## Discussion

A growing body of research has established that removing one eye early in life affords different cortical, functional, and behavioural adaptations compared to people who have had their eye removed late in life, as adults. It is possible that these developmental adaptations contribute to improving future psychosocial experiences related to vision health. Our findings indicate that people who had their eye removed late in life have lower LVQoL compared to binocular viewing control participants. In addition, this finding was associated with self-perception of facial appearance, where lower self-perception of facial appearance is associated with lower LVQoL in this group. Overall, low levels of anxiety and depression were associated with higher perception of facial appearance regardless of whether a participant had an eye removed. Binocular experience and time since enucleation were not related to any psychosocial factor.

People who had one eye removed late in life reported lower LVQoL compared to binocular viewing control participants, unlike people who had an eye removed early in life. In addition, self-perception of facial appearance was also a factor contributing to lower LVQoL but only for participants who had their eye removed late in life. This indicates that there is a distinction between having your eye removed early compared to late in life. Participants in the early monocular enucleation group had their eye removed before 5 years of age where visual disruption occurred during the early critical periods of vision development. It has been established that there are physiological and functional adaptations when one eye is removed early in life (reviewed in [3,5]). These adaptations may impact experience and psychological outlook more broadly in this early monocular enucleation group. This seems plausible since our participants who had an eye removed early in life did not differ on psychosocial factors compared to binocularly intact controls.

In contrast, the average age of eye enucleation of participants who had their eye removed late in life was at approximately 28 years of age – well outside the critical periods of post-natal brain maturation and vision development. A growing body of research shows that monocular enucleation late in life, such as this, is not associated with the same physiological and functional adaptations observed when one eye is removed early in life [7,14]. It appears that the timing of interruption to visual input during post-natal development is a key explanation for this finding. In order to investigate whether this finding was related to developmental factors (such as timing of visual input disruption during development) compared to experiential factors, we assessed time since enucleation (number of months that have passed since removal of one eye) and binocular experience (number of months with binocular vision prior to eye removal). However, we found no relation to the amount of time that had passed since eye enucleation and no relation to binocular experience (time lived with binocular vision). This suggests a greater influence of timing of eye enucleation during development rather than experience with only one eye. Previous studies investigating the impact of removing one eye on psychosocial factors have not distinguished between those who had one eye removed early compared to late in life [16,17,20,25,27,28].

Overall, the current study provides novel insights into factors that contribute to the psychosocial health of people who have had one eye removed, differentiating between those who have had their eye removed early compared to late in life. This may suggest that people who have had one eye removed early in life, during the critical periods of visual development, may benefit from better psychosocial outcomes such as higher LVQoL. Given the nature of this qualitative study and the challenges associated with recruiting patient groups, limitations can be noted. In comparison to qualitative studies of the general population, our study had a relatively small patient sample size (n = 22). The rare nature of eye enucleation poses challenges to recruitment. Additionally, our study was conducted online which limits our ability to provide controlled testing conditions. However, online studies are ideal for rare patient groups who may have accessibility and geographical constraints. It would be valuable for future studies to consider objective measures of participants’ eye prostheses in the event that prosthetic quality is a co-factor for psychosocial health.

In conclusion, our study indicates that developmental factors rather than experiential factors influence psychosocial health in people with one eye. This is reflected in our findings which show that experience with binocularity and time since enucleation are not related to psychosocial health factors. Instead, it is likely that the physiological and functional adaptations, driven by neuroplasticity during the critical periods of visual development, may provide a quality-of-life advantage for individuals who had an eye removed early in life. Our findings underscore the importance of age at which enucleation occurs when considering psychosocial health in people who have had one eye removed in both clinical practice and future research.

## References

[1] Daw NW. Visual development. 4th ed. Boston: Springer. 2014.

[2] Golarai G, Ghahremani DG, Whitfield-Gabrieli S, Reiss A, Eberhardt JL, Gabrieli JDE, et al. Differential development of high-level visual cortex correlates with category-specific recognition memory. Nat Neurosci. 2007;10(4):512–22. doi: 10.1038/nn1865 17351637 PMC3660101

[3] Steeves JKE, González EG, Steinbach MJ. Vision with one eye: a review of visual function following unilateral enucleation. Spat Vis. 2008;21(6):509–29. doi: 10.1163/156856808786451426 19017480

[4] Moshfeghi DM, Moshfeghi AA, Finger PT. Enucleation. Surv Ophthalmol. 2000;44:277–301. 10667436 10.1016/s0039-6257(99)00112-5

[5] Kelly K, Moro S, Steeves J. Living with one eye: plasticity in visual and auditory systems. In: Steeves J, Harris L, editors. Plasticity in sensory systems. Cambridge: Cambridge University Press. 2013. p. 94–108.

[6] Kelly KR, DeSimone KD, Gallie BL, Steeves JKE. Increased cortical surface area and gyrification following long-term survival from early monocular enucleation. Neuroimage Clin. 2014;7:297–305. doi: 10.1016/j.nicl.2014.11.020 25610793 PMC4300017

[7] Kelly KR, McKetton L, Schneider KA, Gallie BL, Steeves JKE. Altered anterior visual system development following early monocular enucleation. Neuroimage Clin. 2013;4:72–81. doi: 10.1016/j.nicl.2013.10.014 24319655 PMC3853349

[8] Moro SS, Steeves JKE. No Colavita effect: equal auditory and visual processing in people with one eye. Exp Brain Res. 2012;216(3):367–73. doi: 10.1007/s00221-011-2940-4 22105335

[9] Moro SS, Steeves JKE. No Colavita effect: increasing temporal load maintains equal auditory and visual processing in people with one eye. Neurosci Lett. 2013;556:186–90. doi: 10.1016/j.neulet.2013.09.064 24103371

[10] Moro SS, Steeves JKE. Intact dynamic visual capture in people with one eye. Multisens Res. 2018;31:675–88. 31264607 10.1163/22134808-20181311

[11] Moro SS, Steeves JKE. Normal temporal binding window but no sound-induced flash illusion in people with one eye. Exp Brain Res. 2018;236(6):1825–34. doi: 10.1007/s00221-018-5263-x 29675714

[12] Wong NA, Rafique SA, Moro SS, Kelly KR, Steeves JKE. Altered white matter structure in auditory tracts following early monocular enucleation. Neuroimage Clin. 2019;24:102006. doi: 10.1016/j.nicl.2019.102006 31622842 PMC6812283

[13] Nicholas JJ, Heywood CA, Cowey A. Contrast sensitivity in one-eyed subjects. Vision Res. 1996;36(1):175–80. doi: 10.1016/0042-6989(95)00119-k 8746251

[14] Moro SS, Qureshi FA, Steeves JKE. Perception of the McGurk effect in people with one eye depends on whether the eye is removed during infancy or adulthood. Front Neurosci. 2023;17:1217831. doi: 10.3389/fnins.2023.1217831 37901426 PMC10603249

[15] Feng Y, Zhou C, Jia R, Wang Y, Fan X. Quality of Life (QoL) and Psychosocial Outcomes in Adult Survivors of Unilateral Retinoblastoma (RB) in China. J Ophthalmol. 2020;2020:4384251. doi: 10.1155/2020/4384251 32280521 PMC7125462

[16] Ford JS, Chou JF, Sklar CA, Oeffinger KC, Novetsky Friedman D, McCabe M, et al. Psychosocial Outcomes in Adult Survivors of Retinoblastoma. J Clin Oncol. 2015;33(31):3608–14. doi: 10.1200/JCO.2014.60.5733 26417002 PMC4622100

[17] Hope-Stone L, Brown SL, Heimann H, Damato B, Salmon P. Two-year patient-reported outcomes following treatment of uveal melanoma. Eye (Lond). 2016;30(12):1598–605. doi: 10.1038/eye.2016.188 27589051 PMC5177756

[18] Scannell O, O’Neill V, Dunne M, Baily C, Salih A, Cunningham M, et al. Quality of Life in Uveal Melanoma Patients in Ireland: A Single-Centre Survey. Ocul Oncol Pathol. 2020;6(2):99–106. doi: 10.1159/000501692 32258017 PMC7109402

[19] Frenkel S, Rosenne H, Briscoe D, Hendler K, Bereket R, Molcho M, et al. Long-term uveal melanoma survivors: measuring their quality of life. Acta Ophthalmol. 2018;96(4):e421–6. doi: 10.1111/aos.13655 29369538

[20] Hope-Stone L, Brown SL, Heimann H, Damato B. Comparison between patient-reported outcomes after enucleation and proton beam radiotherapy for uveal melanomas: a 2-year cohort study. Eye (Lond). 2019;33(9):1478–84. doi: 10.1038/s41433-019-0440-0 30988421 PMC7002659

[21] Klingenstein A, Fürweger C, Mühlhofer AK, Leicht SF, Schaller UC, Muacevic A, et al. Quality of life in the follow-up of uveal melanoma patients after enucleation in comparison to CyberKnife treatment. Graefes Arch Clin Exp Ophthalmol. 2016;254(5):1005–12. doi: 10.1007/s00417-015-3216-7 26573389

[22] van Beek JGM, Buitendijk GHS, Timman R, Muller K, Luyten GPM, Paridaens D, et al. Quality of life: fractionated stereotactic radiotherapy versus enucleation treatment in uveal melanoma patients. Acta Ophthalmol. 2018;96(8):841–8. doi: 10.1111/aos.13823 30284368

[23] McBain HB, Ezra DG, Rose GE, Newman SP; Appearance Research Collaboration (ARC). The psychosocial impact of living with an ocular prosthesis. Orbit. 2014;33(1):39–44. doi: 10.3109/01676830.2013.851251 24205995

[24] Gamba A, Romano M, Grosso IM, Tamburini M, Cantú G, Molinari R, et al. Psychosocial adjustment of patients surgically treated for head and neck cancer. Head Neck. 1992;14(3):218–23. doi: 10.1002/hed.2880140309 1587739

[25] Heindl LM, Trester M, Guo Y, Zwiener F, Sadat N, Pine NS, et al. Anxiety and depression in patients wearing prosthetic eyes. Graefes Arch Clin Exp Ophthalmol. 2021;259(2):495–503. doi: 10.1007/s00417-020-04908-0 32870372 PMC7843548

[26] McCullagh D, Puls N, Beaconsfield M, Dempster M, Jackson J, Williams M. Psychological aspects of living with an artificial eye. Orbit. 2022;41(6):745–50. doi: 10.1080/01676830.2021.2013505 35226567

[27] Ye J, Lou L, Jin K, Xu Y, Ye X, Moss T, et al. Vision-Related Quality of Life and Appearance Concerns Are Associated with Anxiety and Depression after Eye Enucleation: A Cross-Sectional Study. PLoS One. 2015;10(8):e0136460. doi: 10.1371/journal.pone.0136460 26317860 PMC4552790

[28] Brandberg Y, Kock E, Oskar K, af Trampe E, Seregard S. Psychological reactions and quality of life in patients with posterior uveal melanoma treated with ruthenium plaque therapy or enucleation: a one year follow-up study. Eye (Lond). 2000;14(Pt 6):839–46. doi: 10.1038/eye.2000.233 11584839

[29] Cheng GY, Li B, Li LQ, Gao F, Ren RJ, Xu XL, et al. Review of 1375 enucleations in the TongRen Eye Centre, Beijing. Eye (Lond). 2008;22(11):1404–9. doi: 10.1038/sj.eye.6702919 17603465

[30] de Boer MR, Terwee CB, de Vet HCW, Moll AC, Völker-Dieben HJM, van Rens GHMB. Evaluation of cross-sectional and longitudinal construct validity of two vision-related quality of life questionnaires: the LVQOL and VCM1. Qual Life Res. 2006;15(2):233–48. doi: 10.1007/s11136-005-1524-9 16468079

[31] Chen H, Jackson T, Huang X. The Negative Physical Self Scale: Initial development and validation in samples of Chinese adolescents and young adults. Body Image. 2006;3(4):401–12. doi: 10.1016/j.bodyim.2006.07.005 18089244

[32] Bjelland I, Dahl AA, Haug TT, Neckelmann D. The validity of the Hospital Anxiety and Depression Scale. An updated literature review. J Psychosom Res. 2002;52(2):69–77. doi: 10.1016/s0022-3999(01)00296-3 11832252

