# Supplementary material for: Timing of eye removal influences low-vision quality of life and self-perception of facial appearance in people with one eye
Source: PLoS One. 2025 May 15;20(5):e0323603. doi: 10.1371/journal.pone.0323603 (PMC12080766; doi:10.1371/journal.pone.0323603)
Supplement: Attachment — Submitted filename: Kinakool_etal_PLOSONE_R1_ResponseToReviwers.docx [file pone.0323603.s002.docx]

**Timing of eye removal influences low-vision quality of life and self-perception of facial appearance in people with one eye**

Aysha N. Kinakool, Stefania S. Moro, and Jennifer K.E. Steeves

**Journal Requirements:**

1. Please ensure that your manuscript meets PLOS ONE's style requirements, including those for file naming. The PLOS ONE style templates can be found at

<https://journals.plos.org/plosone/s/file?id=wjVg/PLOSOne_formatting_sample_main_body.pdf> and

[https://journals.plos.org/plosone/s/file?id=ba62/PLOSOne_formatting_sample_title_authors_affiliations.pdf](https://track.editorialmanager.com/CL0/https:%2F%2Fjournals.plos.org%2Fplosone%2Fs%2Ffile%3Fid=ba62%2FPLOSOne_formatting_sample_title_authors_affiliations.pdf/1/010f0195a79177d8-b70f938f-0d21-4f06-a4c2-d7a7a699a16d-000000/SVfBTvmyQFEw-qLjvyXuLQ-vNGjcFXsN2sDUB-1qQpk=202)

**Response: This has been completed.**

2. Thank you for stating the following financial disclosure:

“This research was supported by grants from Canada First Research Excellence Fund: Vision Science to Application (VISTA) (CFREF-2015-00013); Natural Sciences and Engineering Research Council of Canada (NSERC) (327588); Canada Foundation for Innovation (CFI) (12807).” Please state what role the funders took in the study.  If the funders had no role, please state: "The funders had no role in study design, data collection and analysis, decision to publish, or preparation of the manuscript." If this statement is not correct you must amend it as needed. Please include this amended Role of Funder statement in your cover letter; we will change the online submission form on your behalf.

**Response: We have updated this information in our cover letter.**

3. When completing the data availability statement of the submission form, you indicated that you will make your data available on acceptance. We strongly recommend all authors decide on a data sharing plan before acceptance, as the process can be lengthy and hold up publication timelines. Please note that, though access restrictions are acceptable now, your entire data will need to be made freely accessible if your manuscript is accepted for publication. This policy applies to all data except where public deposition would breach compliance with the protocol approved by your research ethics board. If you are unable to adhere to our open data policy, please kindly revise your statement to explain your reasoning and we will seek the editor's input on an exemption. Please be assured that, once you have provided your new statement, the assessment of your exemption will not hold up the peer review process.

**Response: Data is published on York University’s Dataverse. It can be found at the following DOI:** [**https://doi.org/10.5683/SP3/ZBFRPC**](https://doi.org/10.5683/SP3/ZBFRPC)

4. Please review your reference list to ensure that it is complete and correct. If you have cited papers that have been retracted, please include the rationale for doing so in the manuscript text, or remove these references and replace them with relevant current references. Any changes to the reference list should be mentioned in the rebuttal letter that accompanies your revised manuscript. If you need to cite a retracted article, indicate the article’s retracted status in the References list and also include a citation and full reference for the retraction notice.

**Response: Our reference list has been reviewed and is complete and correct.**

**Comments to Author:**

**Reviewer #1:** The paper is highly informative and meets the criteria for publication in your journal. It provides valuable insights that will be beneficial for future scientists. Given its significance, I strongly recommend its consideration for publication.

**Response: Thank you for your review!**

**Reviewer #2:** The manuscript addresses important issue related to mononuclear vision and the effect of eye enucleation timing on psychological and social aspects of life. The manuscript is nicely written and advances the knowledge related to post enucleation experience in humans. Only one point to be considered by authors, please provide full demographic data of the study subjects (sex, ethnicity, etc.). This will give full clarity to the research and enhance it global exposure.

**Response: We have added a table with demographic data to our manuscript.**
